# Dr. Brown-Sequards’ Elixir

**Published:** 1889-09

**Authors:** 


### DR. BROWN-SEQUARD'S ELIXIR.

AUGUSTA, GA., Aug. 19, 1889.

*To The Editors of The Atlanta Medical and Surgical Journal:*

How in the name of common sense is it possible that a physician and physiologist of the reputation and standing of Dr. Brown-Sequard can come before the medical profession with claims for this so-called Elixir of Life. That coming from an obscure source would at once be condemned as visionary and a piece of quackery.

His whole life-long teaching of physiology is enough to show the irrationality of such a claim; and all the known laws of absorption and nutrition show its want of a "*raison d'être.*"

The use of this elixir in practice will soon show it to be a dangerous and mischievous thing; even if used immediately after its preparation and in a perfectly fresh state, it may, if not at once absorbed decompose in the body, and produce blood poisoning and death.

A few coroner's inquests will put an end to its use.

I am yours, etc.,
R. O. G

